# Assay of the Multiple Energy-Producing Pathways of Mammalian Cells

**DOI:** 10.1371/journal.pone.0018147

**Published:** 2011-03-24

**Authors:** Barry R. Bochner, Mark Siri, Richard H. Huang, Shawn Noble, Xiang-He Lei, Paul A. Clemons, Bridget K. Wagner

**Affiliations:** 1 Biolog, Inc., Hayward, California, United States of America; 2 Chemical Biology Program, Broad Institute of Harvard and MIT, Cambridge, Massachusetts, United States of America; Paris Institute of Technology for Life, Food and Environmental Sciences, France

## Abstract

**Background:**

To elucidate metabolic changes that occur in diabetes, obesity, and cancer, it is important to understand cellular energy metabolism pathways and their alterations in various cells.

**Methodology and Principal Findings:**

Here we describe a technology for simultaneous assessment of cellular energy metabolism pathways. The technology employs a redox dye chemistry specifically coupled to catabolic energy-producing pathways. Using this colorimetric assay, we show that human cancer cell lines from different organ tissues produce distinct profiles of metabolic activity. Further, we show that murine white and brown adipocyte cell lines produce profiles that are distinct from each other as well as from precursor cells undergoing differentiation.

**Conclusions:**

This technology can be employed as a fundamental tool in genotype-phenotype studies to determine changes in cells from shared lineages due to differentiation or mutation.

## Introduction

Tetrazolium-based redox assays can be used to measure the active *in vivo* energy-producing pathways of a very wide range of microbial cells [1], [2], including eukaryotic microbial cells that undergo mitochondrial respiration. Tetrazolium reduction is a ubiquitous property of cells, enabling a simple colorimetric cellular assay technology to be multiplexed into thousands of different assays and facilitating detailed cellular analyses in what we term Phenotype MicroArrays [3]–[6]. Based on previous success in profiling metabolic activity of microbial cells (www.biolog.com/mID_section_13.html), we sought to develop a redox chemistry that could extend comparable analytical capabilities to mammalian cells.

Redox dyes such as tetrazolium dyes (*e.g.*, MTT, MTS, XTT) and resazurin-based dyes (*e.g.*, Alamar Blue) measure nonspecific cellular reductase activities [7]–[10]. We assayed various mammalian cell lines in RPMI-1640 medium without carbon energy sources (glucose and glutamine) and found substantial levels of non-specific background dye reduction, which was exacerbated by adding serum or electron carriers; however, we were able to develop two redox chemistries (Dye Mix MA and MB) that give very little non-specific dye reduction (see Figure S1 and Figure S2) and are reduced by a wide range of mammalian cells. Importantly, the reduction of these dyes is dependent on the presence of a usable carbon-energy source in the medium, such as glucose. Therefore, just as in microbial cells, our new redox dye chemistries measure reductase activity due to energy (NADH) producing catabolic pathways that use diverse biochemical substrates.

The extent to which animal cells from various organs and tissues use different carbon substrates for energy has not been systematically investigated. It is known that, in addition to glucose, animal cells can metabolize and grow on other substrates [11]–[19]. A survey of nutrient metabolism [12] found 32 carbohydrates that could be metabolized by mammalian cells, of which 15 could support the growth of at least one of five cell lines tested. However, as *in vitro* culture media were developed using glucose, pyruvate, and glutamine as energy sources, which were shown to support growth of most cells of interest, the impetus to study further the scope and diversity of nutrients metabolized by different cell types waned. Information about all pathways that contribute to energy production not only underlies our fundamental understanding of animal nutrition, but also is likely to help us understand and treat undesirable or aberrant metabolism, such as in obesity and diabetes. It may also help us develop new approaches in nutritional therapy to improve treatment of a wide range of conditions, including aging, cancer, infectious disease, inflammation, and wound repair [20].

## Results

### Metabolic fingerprinting of cancer cells

**Figure 1:**
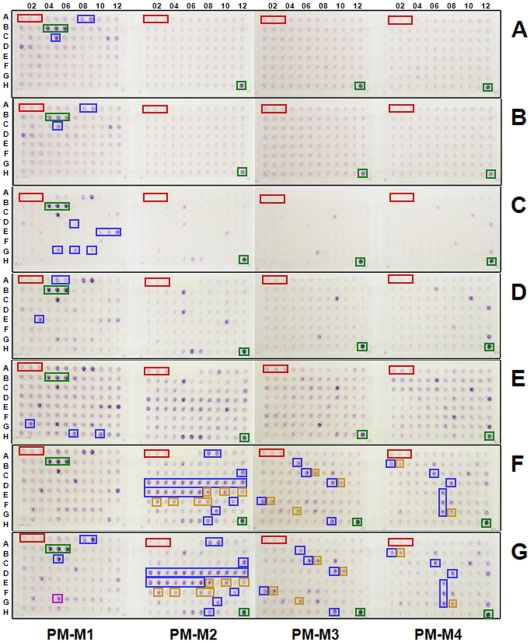
Assay of seven human cell lines in 367 metabolic assays. Suspensions of the following cancer cells were inoculated into Phenotype MicroArrays PM-M1 through PM-M4 (Biolog, Hayward, CA) which contained 367 biochemical substrates that could potentially be metabolized and provide energy for cells: (**A**) CCRF-CEM leukemia, (**B**) HL-60 leukemia, (**C**) PC-3 prostate cancer, (**D**) A549 non-small cell lung cancer, (**E**) COLO205 colon cancer, (**F**) HepG2 hepatocellular cancer, (**G**) HepG2/C3A, a clonal variant of HepG2. Adherent cells were washed twice in Dulbecco's PBS (D-PBS) before being detached with trypsin. Detached cells and non-adherent cells were suspended at 400,000 cells/mL in RPMI-1640 that lacked phenol red and glucose and was supplemented with PS and reduced levels of glutamine (0.3 mM) and FBS (5%). The cells were dispensed at 50 µL per well (20,000 cells per well) into the four microplates. Plates were incubated for 40 hr at 37°C under 5% CO_2_-95% air. This 40-hour incubation allows cells to use up residual carbon-energy sources in the 5% serum (*e.g.*, 5% serum would contribute about 0.35 m*M* glucose, plus lipids, and amino acids) and minimizes the background color in the negative control wells which have no added biochemical substrate. Furthermore, the 40-hour incubation allows cells to transition their metabolism to use the various substrates provided in the wells. After incubation, we added 10 µL of 6-fold concentrated Redox Dye Mix MA (Biolog, Hayward, CA) to HepG2/C3A, HepG2, COLO 205, A549, PC-3 cells whereas Redox Dye Mix MB was added to HL-60 and CCRF-CEM cells. Plates were incubated at 37°C under air to assess dye reduction for 5 hr (Redox Dye Mix MA) or 24 hr (Redox Dye Mix MB) and then photographed. Negative controls (red boxes) have no substrate in the well. Wells containing D-glucose (green boxes) serve as a positive control. Additional differentially metabolized substrates (blue boxes) for each cell line are described in the text.

To demonstrate the application of this platform to human cells, we profiled seven diverse human cancer cell lines in four microplates (termed PM-Ms) containing 367 substrate nutrients (Figure 1; see Table S1 for a complete map of the nutrients). PM-M1 contains primarily carbohydrate and carboxylate substrates, whereas PM-M2, -M3, and -M4 contain individual L-amino acids and most dipeptide combinations. With the exception of abiotic color formation in a few wells containing reducing sugars (palatinose, D-turanose, D-tagatose, and L-sorbose), multiple lines of evidence presented below suggest that the color formed from each substrate reflects the energy-producing activity of its catabolic pathway. This method is simple to perform and reproducible (Figure S3).

All cell lines tested produced a strong reductive response in wells containing glucose (Figure 1; green boxes, all panels) and little or no response in wells lacking any carbon source (Figure 1; red boxes, all panels). Two leukemic cell lines (CCRF-CEM and HL-60) show additional metabolism limited to D-mannose, D-maltose, and maltotriose (Figure 1A,B; blue boxes). Prostate cancer cells (PC-3) show additional metabolism of fructose, some nucleosides, and tricarboxylic acid (TCA) cycle intermediates (Figure 1C; blue boxes), and lung cancer cells (A549) show additional metabolism of dextrin, glycogen, and galactose (Figure 1D; blue boxes). In addition, both prostate and lung cells show metabolism of glutamine and many glutamine-containing dipeptides (Figure 1C,D; purple wells on PM-Ms 2-4). Both colon (COLO205) and liver (HepG2) cancer cell lines demonstrate a more diverse catabolic response, with more wells showing a strong reductive response (Figure 1E–G). For example, colon cells additionally metabolize lactic acid, butyric acid, and propionic acid (Figure 1E; blue boxes), and liver cells metabolize alanine, arginine, and dipeptides containing them (Figure 1F,G; Ala = blue boxes, Arg = gold boxes). This last result is consistent with an NMR-based report that alanine is elevated in response to hypoxia, and is associated with hepatomas [21].

Importantly, we observe that two genetically related liver cell lines have similar metabolic profiles. The HepG2/C3A cell line (Figure 1G) was derived as a spontaneous clonal variant of HepG2 (Figure 1F), selected for its ability to grow on pyruvate as the principal energy source [22]. Accordingly, this clone shows stronger metabolism of pyruvate (Figure 1G; magenta box) relative to its parent. Interestingly, along with this metabolic change, HepG2/C3A cells have also been reported to have additional phenotypes more typical of normal liver cells, such as producing and secreting higher levels of albumin and other serum proteins [22]. The colon cell line COLO205 has a similar metabolic profile to HepG2, with a salient difference being the observed increased metabolism of butyric acid and propionic acid, which are plentiful in the colonic environment due to their production by resident anaerobic bacteria. Thus, the substrate utilization pattern provides a metabolic characterization as well as a unique profile of the metabolism of an animal cell.

These results with human cell lines mirror our results with bacterial and fungal cells [2], in that we observe differential catabolic activities in different cell types consistent with the known physiological properties of different cells. Furthermore, they recapitulate the results of earlier studies [12] that showed five different cell lines were readily distinguishable by their profiles of carbohydrate preference. The substrate nutrients in PM-M1 to –M4 are all present at low millimolar concentrations. Analyses of blood chemistries from non-fasting normal humans suggest that the principal circulating carbon energy sources are glucose (2–10 m*M*) and fatty acids, both free (0.01–1.0 m*M*) and in the form of triglycerides and lipoproteins [23], [24]. Glutamine (0.6 m*M*), alanine (0.35 m*M*), and lactic acid (2 m*M*) also have significant levels in normal blood [24], [25]. A recent metabolomic study has documented a wider array and higher concentrations of potentially metabolizeable biochemicals in human plasma after exercise, including glucose-6-phosphate, inosine, uridine, alanine, lactate, pyruvate, succinate, malate, fumarate, and glycerol [26]. Furthermore, the cells lining the intestine are exposed to an even more diverse and variable nutritional environment. Similarly, the liver is fed directly from the intestine by the portal circulation. Therefore, it is reasonable to expect that colon and liver cells would have more diverse capabilities for substrate metabolism than cells from other tissues.

### Mechanism of cellular dye reduction

**Figure 2:**
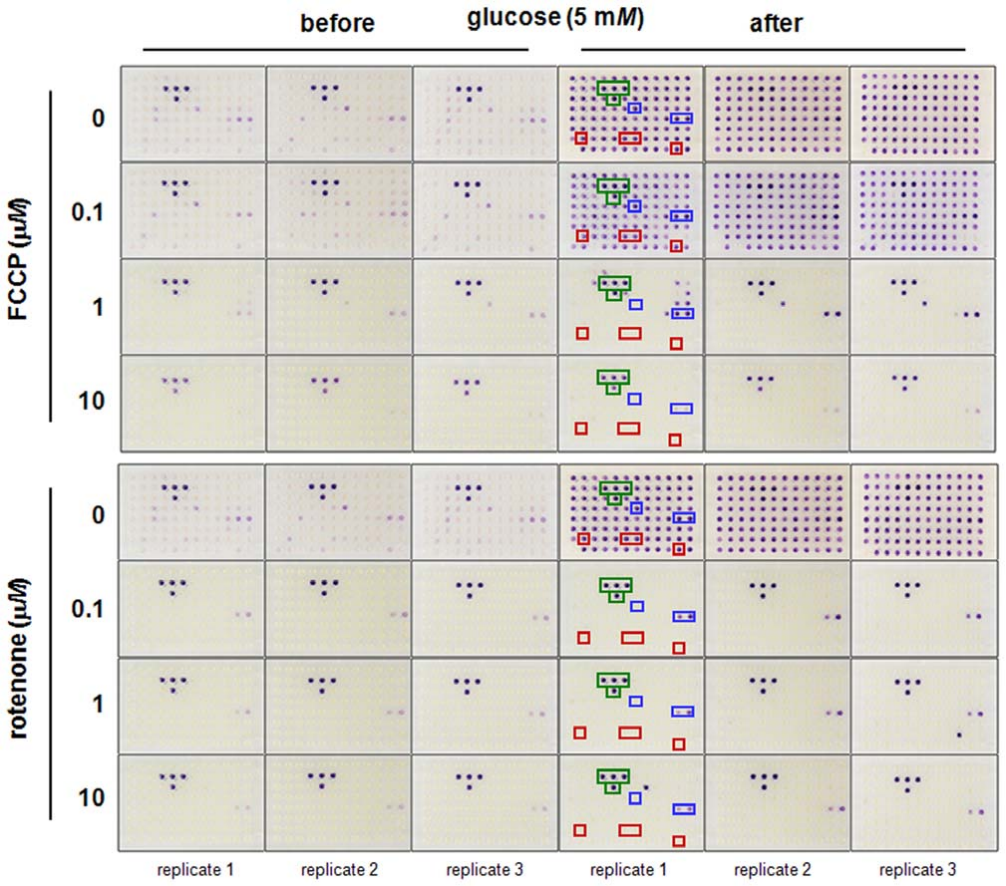
Effect of mitochondrial inhibitors on energy-producing pathways of HepG2/C3A cells. HepG2/C3A cells were washed twice with serum-free inoculating medium (RPMI-1640 with no phenol red or glucose but containing PS, 0.3 m*M* glutamine) and resuspended in the same medium at a density of 400,000 cells/mL with varying concentrations of either FCCP or rotenone. Control cells were incubated with 0.003% DMSO, the solvent for both FCCP and rotenone. These cell suspensions were immediately inoculated into PM-M1 at 50 µL per well. After an 18-hour incubation at 37°C under 5% CO_2_-95% air, 10 µL of Redox Dye Mix MA was added to each well. After 5 hours with the dye, the plates were photographed. After the plates were photographed, glucose was added to each well of the plates to a final concentration of 5 m*M* to assess cell viability. The plates were then incubated overnight and photographed again.

To study further the mechanistic aspects of this dye-reduction assay, we tested HepG2/C3A cells in PM-M1 panels with varying concentrations of the mitochondrial inhibitors carbonyl cyanide-*p*-trifluoromethoxyphenylhydrazone (FCCP) and rotenone. Even at low concentrations, these inhibitors completely block dye reduction by substrates that would presumably be oxidized mitochondrially, such as lactic acid, α-keto-glutaric acid, succinamic acid, and acetic acid (Figure 2; red boxes). At higher concentrations, they partially block reduction of fructose, adenosine, and inosine (Figure 2; blue boxes), but have little effect on color formation in wells containing glucose or mannose (Figure 2; green boxes). Adding back 5 mM glucose after an 18-hour exposure is not sufficient to rescue the cells when the concentration of the inhibitor was high enough to block substrate catabolism. This result is consistent with our interpretation that the assay reflects the production of NADH from various substrates, and that the fraction of NADH produced mitochondrially is different for different energy substrates.

**Figure 3:**
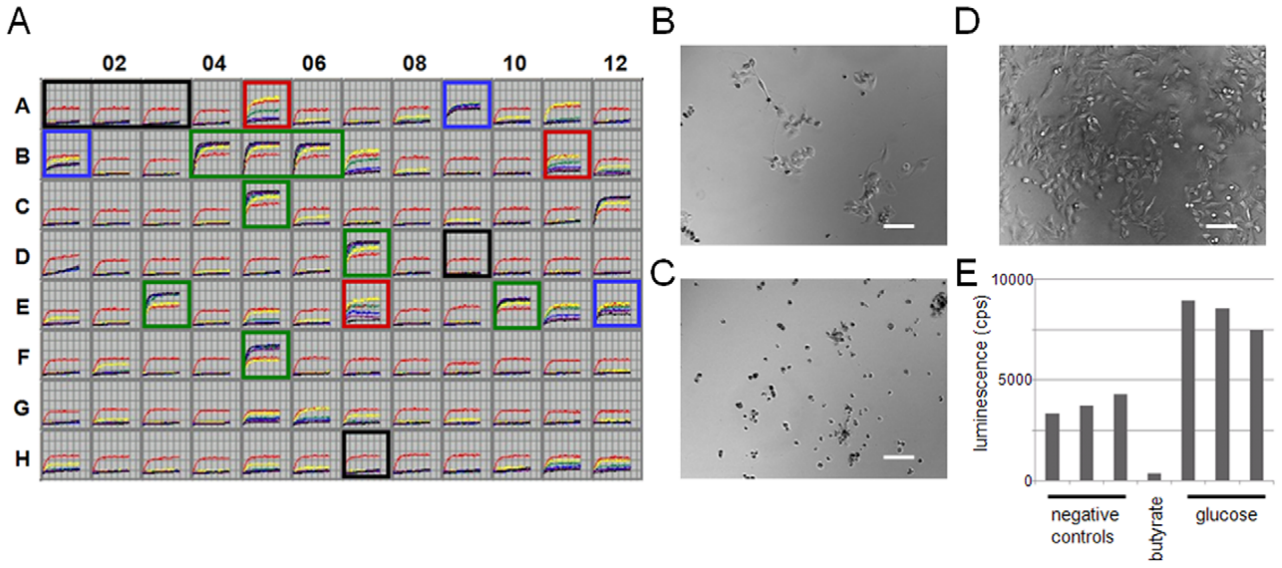
Assay of growth, stasis, or death under different nutritional conditions. (**A**) HepG2/C3A were suspended at 50,000 cells per mL in serum-free RPMI-1640 medium that lacked phenol red and glucose, but contained PS and 4 mM glutamine. Cells were dispensed into six PM-M1 microplates at 50 µL per well (2,500 cells per well) and incubated at 37°C under 5% CO_2_-95% air. On six consecutive days, starting on day 0 when the cells were plated, 10 µL of Redox Dye Mix MA containing 30 m*M* glucose (a 6-fold concentrate) was added to one microplate, the plate was sealed with tape (LMT-SEAL-EX, Phenix Research Products, Hayward, CA) to prevent CO_2_ loss, and incubated at 37°C in an OmniLog instrument (Biolog, Hayward, CA) for 18 hours to kinetically record formation of purple formazan in the wells on days 0 (red) 1 (yellow) 2 (green) 3, (blue) 4 (purple) and 5 (black). Substrates resulting in distinguishable responses are indicated: cell proliferation (green boxes), cell stasis (blue boxes), slow cell death (red boxes), rapid cell death (black boxes). In these kinetic graphs, the X-axis is time and the Y-axis is OmniLog color density units. Additional details on the testing protocol, OmniLog instrument, and kinetic assay reproducibility are provided in Figure S3. (**B**) Microscopic image of HepG2 in a negative-control well after three days in culture. (**C**) Microscopic image of HepG2 in a butyrate-containing well after three days in culture. (**D**) Microscopic image of HepG2 in a D-glucose-containing well after three days in culture. (**E**) Quantification of ATP levels in HepG2 cells after three days in culture, using a luciferase-based method. Scale bars  = 100 µm.

The ability of a cell to use a particular nutrient as a source of energy is necessary but not sufficient to use it as a growth substrate. To use a chemical as a sole growth substrate, the cell must also be able to convert the carbon skeleton of that chemical into all other metabolites that it requires. This assay format can also be used to determine the survival responses of cells under different conditions of substrate supply. HepG2/C3A cells were tested for cell growth or death with PM-M1 substrates over a five-day incubation (Figure 3A). Four qualitatively distinguishable responses were observed: proliferation (Figure 3; green boxes), including wells containing D-glucose, D-mannose, D-fructose, D-galactose, uridine, and xylitol; stasis (Figure 3A; blue boxes), including wells containing D-maltose, D-glucose-6-phosphate, and inosine; slow death (Figure 3A; red boxes), including wells containing dextrin, D-sorbitol, and pectin; and rapid death, including wells corresponding to no substrate, D-raffinose, and butyrate (Figure 3A; black boxes). Sodium butyrate, a general inhibitor of histone deacetylases, has previously been shown to be quite toxic to HepG2 cells [27]. The set of substrates assayed as positive for energy production corresponds with substrates supporting prolongation of survival, as corroborated by microscopic observation of the healthy morphology and increased cell numbers in these wells (Figure 3D, compared to Figures 3B, C). Further, the quantification of ATP levels in these wells, after three days in cell culture, shows consistency with our method (Figure 3E). Thus, the ability to produce energy from a substrate corresponds with increased survival, even if only temporarily. One possible exception is pectin which may slow cell death by a mechanism other than energy production.

### Metabolic profiling of adipocytes

We sought to apply this technology to a cellular model system representing a highly metabolically active state. We reasoned that adipocyte biology would be well-suited for nutrient-response profiling, since we could compare white and brown adipocytes, which have very different physiological roles, as well as analyze the cell-state changes that must occur during differentiation of preadipocyte fibroblasts. Therefore, we profiled the mouse cell line 3T3-L1, which has been used for decades as a model of white fat [28], and an immortalized brown preadipocyte cell line [29], in both undifferentiated and differentiated states. This experimental design enabled us to focus on four major comparisons: brown preadipocytes with brown adipocytes, white preadipocytes with white adipocytes, brown with white preadipocytes, and brown with white adipocytes.

**Figure 4:**
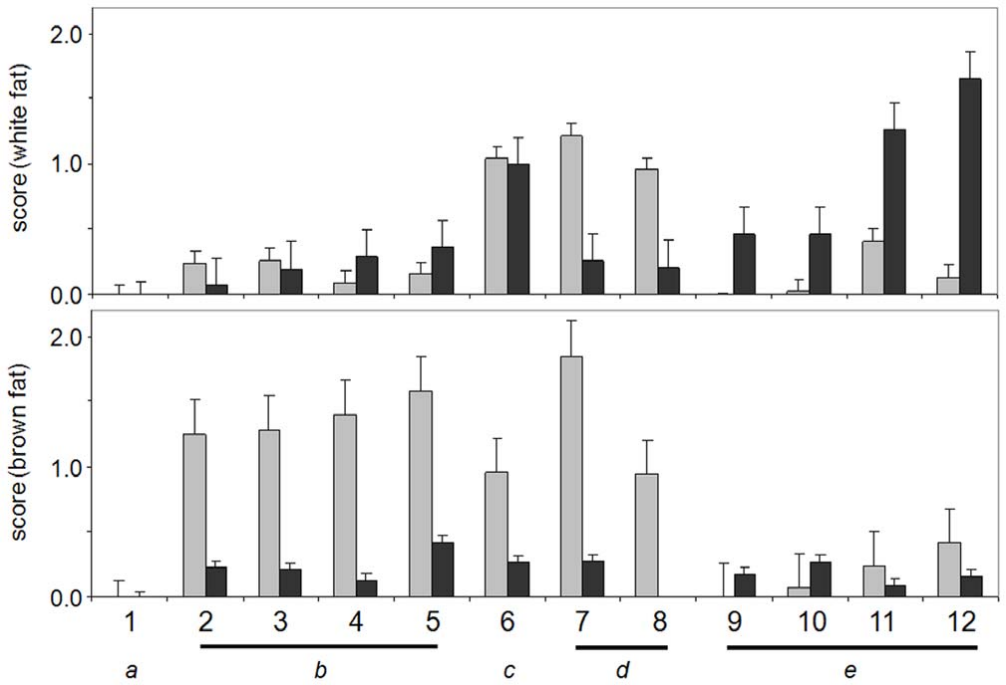
Comparison of substrate metabolism in brown and white preadipocytes and adipocytes. Normalized optical-density scores representing nutrient metabolism, showing the greatest differences between white fat (top) and brown fat (bottom) cells in either an undifferentiated (gray bars) or differentiated (black bars) state. Included are a) wells that cause no change (1, negative control), b) nutrients that are high in brown preadipocytes and decrease upon differentiation (2, α-D-glucose-1-phosphate; 3, D-glucose-6-phosphate; 4, D-fructose-6-phosphate; 5, glycogen), c) a similarly behaved nutrient that is high in white fat (6, hexanoic acid), d) nutrients that decrease during differentiation of both cell types (7, dextrin; 8, butyric acid), and e) nutrients that increase upon white fat differentiation (9, L-leucine; 10, Leu-Leu; 11, succinamic acid, 12. acetoacetic acid). The normalized scores shown are reprentative of two independent biological experiments. The error bars represent the error for the six alpha-D-glucose wells in each experiment, which was taken as the largest and most conservative estimate of the error for other nutrients.

In order to quantitatively compare each state in a rapid and systematic manner, we developed a scoring system based on the optical density of each well at 590 nm, normalized to negative-control wells. As expected, white and brown preadipocytes and adipocytes have different metabolic profiles as demonstrated with this dye-reduction assay (Figure 4). In order to quantify these comparisons, we also calculated a comparison score (CS) as the absolute value of the ratio between two states being compared at a particular time point (Figure S4). Quantitatively, the greatest difference between brown and white preadipocytes was in their metabolism of glycogen, with brown preadipocytes showing faster metabolism (Figure S4 and Figure S5). We also observed much stronger metabolism of the three sugar phosphates (fructose-6-phosphate, glucose-1-phosphate, and glucose-6-phosphate) by brown preadipocytes (Figure 4). Interestingly, the metabolism of these four nutrients decreased during brown adipogenesis.

We also sought to identify differences in substrate metabolism that might occur during adipocyte differentiation (Figure 4). Surprisingly, all changes that occurred in brown adipocytes after differentiation were decreases in metabolism. Like 3T3-L1 differentiation, brown adipocyte differentiation resulted in a decreased metabolism of dextrin and butyric acid. On the other hand, after differentiation, there were more substrates for which dye reduction increased in white and not brown adipocytes. Acetoacetic acid and succinamic acid resulted in some the greatest increases in dye reduction. Indeed, for adipocytes, acetoacetic acid consistently produced the strongest dye reduction of any substrate tested, including D-glucose. Remarkably, all peptides containing leucine resulted in at least some increase in metabolism after white adipogenesis, although at a low absolute rate of dye reduction (see Leu and Leu-Leu, Figure 4). These results indicate that, while these adipogenic processes may superficially appear to be similar, each produces a different metabolic response.

## Discussion

We have developed a simple colorimetric assay method for simultaneously measuring multiple energy-producing pathways in cells and providing a more global perspective of the range and regulation of multiple energy-producing pathways. This provides a new tool for researchers wanting to study metabolic pathway activities, with advantages for measuring *in vivo* metabolic fluxes. Metabolomic assay of pool levels are better for detecting the presence of pathways and detecting the site of pathway blockages, but changes in pathway fluxes may not result in increases or decreases of pool levels. Another approach to measuring pathway fluxes using quantitative isotope tracking, relies on detailed and accurate knowledge of the pathways present and active. This new colorimetric PM assay is not only extremely simple to perform, but it does not require any prior knowledge of the energy pathways present and active in a cell line.

Each well in the PM assay panel provides both qualitative and quantitative data on the potential of a cell line to metabolize a biochemical substrate and produce energy, presumably as NADH. We have provided evidence that glucose is not the only relevant energy source used by colon cells, liver cells, and adipocytes. This is likely to be the case for other cells as well. Muscle cells [30] and brown fat cells [31] are known to actively metabolize fatty acids. During periods of exercise, blood concentrations of glycerol (20-120 µ*M*), pyruvic acid (40-180 µ*M*), and lactic acid (0.5–5 m*M*), rise to significant levels [26], [32]. To prevent the accumulation of toxic levels of acids in muscles, lactic acid is converted *via* pyruvic acid to alanine, which is subsequently metabolized, primarily by the liver [33], [34]. The dominant view has been that alanine is primarily converted to glucose rather than oxidized to CO_2_. Our survey of seven cell lines confirms that liver cells are especially proficient in metabolism of alanine, but also shows that this amino acid can be used to produce energy. These results are consistent with metabolomic profiling methods [21], showing that this simple method provides an independent, flux-based measurement of cellular metabolism. Under nutritional stress conditions such as starvation, acetoacetic acid and D-β-hydroxy-butyric acid can become principal energy sources for cells [35]. Further, under long-term calorie-restricted diets, pyruvate may play a key role both as a fuel for mitochondrial energy production and as a regulator of the SIRT1 protein in hepatocytes [36]. This evidence suggests the importance of broadening the study of energy metabolism beyond glucose.

Liver cells, enteroendocrine cells, and adipocytes seem particularly important to study in the context of multiple energy-metabolism assays, obesity, and diabetes. The liver plays an essential physiological role in helping to maintain a constant supply of glucose and other substrates to the rest of body. Our method confirms previous reports showing the toxic effect of sodium butyrate on HepG2 cells [27]. Enteroendocrine cells of various types are present in the gut epithelium, sensing certain substrates in the lumen (*e.g.*, methyl-pyruvate, arginine, peptides) and releasing incretin hormones such as glucose-dependent insulinotropic peptide, glucagon-like peptide 1, ghrelin, and cholecystokinin, which affect appetite, metabolism, and energy storage by a variety of mechanisms [37], [38]. Adipocytes not only manage the storage of excess energy in the body, but also produce and release a number of adipokine-signaling hormones [39].

We observed several significant differences in dye-reduction profiles between white and brown adipocytes. For example, the metabolism of glycogen was high in brown *versus* white preadipocytes, but decreased during cellular differentiation. This observation is consistent with a report that murine brown preadipocytes show a myogenic transcriptional profile, suggesting that brown and white fat arise from separate cell lineages [40]. More recently, the transcription factor PRDM16 has been shown to control cell fate in mice; overexpression in myoblasts results in a brown fat state, while loss of expression induces muscle differentiation [41]. Another consistent difference between brown and white preadipocytes was the elevated metabolism of various sugar phosphates. These results support previous literature reports, in which brown adipose tissue was shown to have a 15-fold increase in glucose 6-phosphate dehydrogenase activity [42]. Our results provide additional evidence for the relationship between brown preadipocytes and muscle, and show that this assay technology can be used to examine similarities between distinct cell types, and to dissect subtle differences between cell types thought to be similar.

In addition to using this assay technology to examine and compare metabolic differences in cells from various tissues, it is also productive to compare isogenic cell lines with specifically-engineered mutations. One of the most significant aspects of this work is the metabolic comparison of the nearly isogenic cell lines HepG2 and HepG2/C3A. The C3A line was selected by its ability to grow on pyruvate, and our assay showed that increased pyruvate metabolism was the salient detectable metabolic phenotype. With this tool, it would be illuminating to examine changes in global energy-producing metabolism in mammalian cells with knockouts or activations of key genes, for example those encoding p53, Foxo3a, SIRT1 [43], ChREBP [44], BAD [45], LXR [46], PPARδ [47], GCN 5, or PGC-1α [48]. The assay technology could also be used in conjunction with RNAi gene silencing studies in cell lines or primary cells.

Other areas where metabolic profiling analysis of multiple energy-producing pathways may lead to advancement are cancer, nutrition, and aging. For example, there are potential applications in studying the energy metabolism of non-glucose compounds in PET imaging in neurology and cancer diagnostics. There is renewed interest in the Warburg effect [49] and in using it in novel approaches to killing cancer cells with energy antagonists such as 2-deoxyglucose [50] or Akt inhibitors [51]. Energy metabolism is also relevant in calorie-restricted longevity and cachexia. This method provides biologists with a simple tool for precise and detailed phenotypic analysis of animal cells and, as we have previously shown with microbial cells [6], enables a wide range of studies relating genotype to phenotype.

## Materials and Methods

### Cell culture

All cell lines were obtained from ATCC (Manassas, VA). Cells were cultured in an RPMI-1640-based media (without phenol red, with penicillin/streptomycin and 10% FCS) and incubated at 37°C with 6.5% CO_2_. Cells were washed in Dulbecco's PBS and resuspended at a density of 400,000 cells/mL in matched medium depleted of carbon-energy sources (no glucose, low glutamine (0.3 m*M*), and low FCS (5%)). 50 µL per well of this suspension (20,000 cells per well) is dispensed into four microplates containing 367 biochemicals that could potentially be metabolized and provide energy for cells. We found that a 40-hour incubation is optimal for decreasing the background color in the negative control wells and increasing the color in various wells with metabolized substrates, to allow cells to use any remaining carbon-energy sources (*e.g.*, from the glucose (5% serum would contribute about 0.35 m*M* glucose), lipids, glutamine, and other amino acids in the serum), and to adapt the cells to using the substrate provided in the well. After this incubation, 10 µL of Redox Dye Mix MA (HepG2/C3A, HepG2, COLO 205, A549, PC-3) or MB (HL-60 and CCRF-CEM) was added, and cells were incubated to assess dye reduction for 5 hours (Redox Dye Mix MA) or 24 hours (Redox Dye Mix MB) and photographed.

### Effect of mitochondrial inhibitors in HepG2/C3A cells

HepG2/C3A cells were washed twice with serum-free inoculating medium (modified RPMI 1640, with 1X vitamins, 1X salts, 1X amino acids, 0.3 m*M* glutamine, and no glucose) and resuspended in the same medium at a density of 400,000 cells/mL with varying concentrations of either FCCP or rotenone. Control cells were incubated with 0.003% DMSO, the solvent for both FCCP and rotenone. These cell suspensions were immediately inoculated into PM-M1. After an 18-hour incubation, 10 µL of Redox Dye Mix MA was added to each well. After 5 hours with the dye, the plates were photographed. After the plates were photographed, glucose was added to each well of the plates to a final concentration of 5 m*M* to assess cell viability. The plates were then incubated overnight and photographed again.

### Assessment of cell proliferation, stasis, or death under different nutritional conditions

HepG2/C3A cells were suspended in inoculating medium with 4 m*M* glutamine and 1X penicillin/streptomycin, seeded at 2500 cells per well into six PM-M1 microplates, and incubated at 37 C with CO_2_. Each day, one microplate was removed and Biolog Redox Dye MA plus 5 m*M* glucose was added. The microplate was then incubated in an OmniLog for 18 hours with purple color formation data recorded. Cellular ATP levels were measured using the luciferase-based CellTiterGlo kit (Promega).

### Adipocyte cell culture

3T3-L1 preadipocytes were grown in Dulbecco's Modified Eagle's Medium (DMEM) supplemented with 10% donor calf serum and 100 µg/mL penicillin/streptomycin mix in a humidified atmosphere at 37C with 5% CO_2_. Immortalized brown preadipocytes were cultured similarly, in media containing 10% fetal bovine serum (FBS). Adipocytes were differentiated prior to incubation in PM-M microplates, such that >90% of cells contained lipid droplets. 3T3-L1 cells were differentiated by culturing 48 hours in DMEM with 10% FBS, 170 n*M* insulin, 2 µg/mL dexamethasone, and 250 µ*M* isobutylmethylxanthine (IBMX), followed by changing the media every 48 hours to DMEM containing FBS and insulin. Brown preadipocytes were differentiated by culturing 48 hours in DMEM with 10% FBS, 20 n*M* insulin, 250 µ*M* indomethacin, 1 n*M* triiodothyronine (T3), 2 µg/mL dexamethasone, and 250 µ*M* IBMX, followed by changing the media every 48 hours to DMEM containing FBS, insulin, and T3.

## Supporting Information

Figure S1 **A549 lung cells were cultured as described in** **Materials and Methods** **, suspended in RPMI-1640 medium without phenol red, and 50 µL of cell suspension was added to each column of wells at 2-fold dilutions (right to left) in the same medium.** After 4 hours, 10 µL of 4 different redox dye chemistries were added to a final tetrazolium concentration of 500 µM.
(TIF)

Figure S2 **HepG2/C3A liver cells were cultured in Phenotype MicroArray PM-M1 for 40 hours and assayed for dye reduction, as described in** **Materials and Methods** **.** Four different redox dye chemistries were used but in all cases, the concentration of the tetrazolium dye was 500 µM.
(TIF)

Figure S3 **PM Assay with kinetics of dye reduction recorded by the OmniLog instrument.** This figure shows the steps in running a PMM assay. Assays are initiated by adding a cell suspension to the wells, followed by addition of a redox dye. The PMM microplates are placed inside of the OmniLog instrument, which incubates the microplates and reads the color formation in wells with an internal color video camera every 15 minutes. The OmniLog software then generates kinetic graphs of color versus time for all wells. These assays have very high reproducibility. In the example shown, HME human breast cells (a generous gift of Dr. Chris Torrance, Horizon Discovery Ltd., Cambridge, UK) were tested using the standard protocol, but without serum and with the glutamine concentration increased to 2 mM. The graph shows a triplicate repeat of the assay with runs shown in green, red, and black.
(TIF)

Figure S4 **Comparison of substrate metabolism in brown and white preadipocytes and adipocytes.** All adipoctye cell lines were assayed with Redox Dye Mix MB. (**A**) Substrates resulting in the greatest difference in comparison score (CS) at 24 hours between immortalized brown preadipocytes (blue lines) and 3T3-L1 white preadipocytes (green lines). To simplify the analysis, we only considered the CS at the 24-hour time point measurement, reasoning that since the accumulation of signal only increases over time, we would detect the most stable differences at this time, rather than only detecting kinetic changes in absorbance. (**B**) Substrates resulting in the greatest difference in CS at 24 hours between fully differentiated brown (blue lines) and 3T3-L1 white adipocytes (green lines). Graphs are depicted as the normalized change in absorbance (ΔΔAbs), using the zero-hour time point as baseline.
(TIF)

Figure S5 **Analysis of effects of adipocyte differentiation on substrate metabolism.** (**A**) Substrates resulting in the greatest difference in comparison score (CS) at 24 hours between undifferentiated 3T3-L1 preadipocytes (blue lines) and 3T3-L1 white adipocytes (green lines). (**B**) Substrates resulting in the greatest difference in CS at 24 hours between undifferentiated brown preadipocytes (blue lines) and brown adipocytes (green lines). Graphs are depicted as the normalized change in absorbance.
(TIF)

Table S1 **Plate maps of Phenotype MicroArray MicroPlates PM-M1 through -M4.**
(DOC)
