# Supplementary figures and images for: Assay of the Multiple Energy-Producing Pathways of Mammalian Cells
Source: PLoS One. 2011 Mar 24;6(3):e18147. doi: 10.1371/journal.pone.0018147 (PMC3063803; doi:10.1371/journal.pone.0018147)

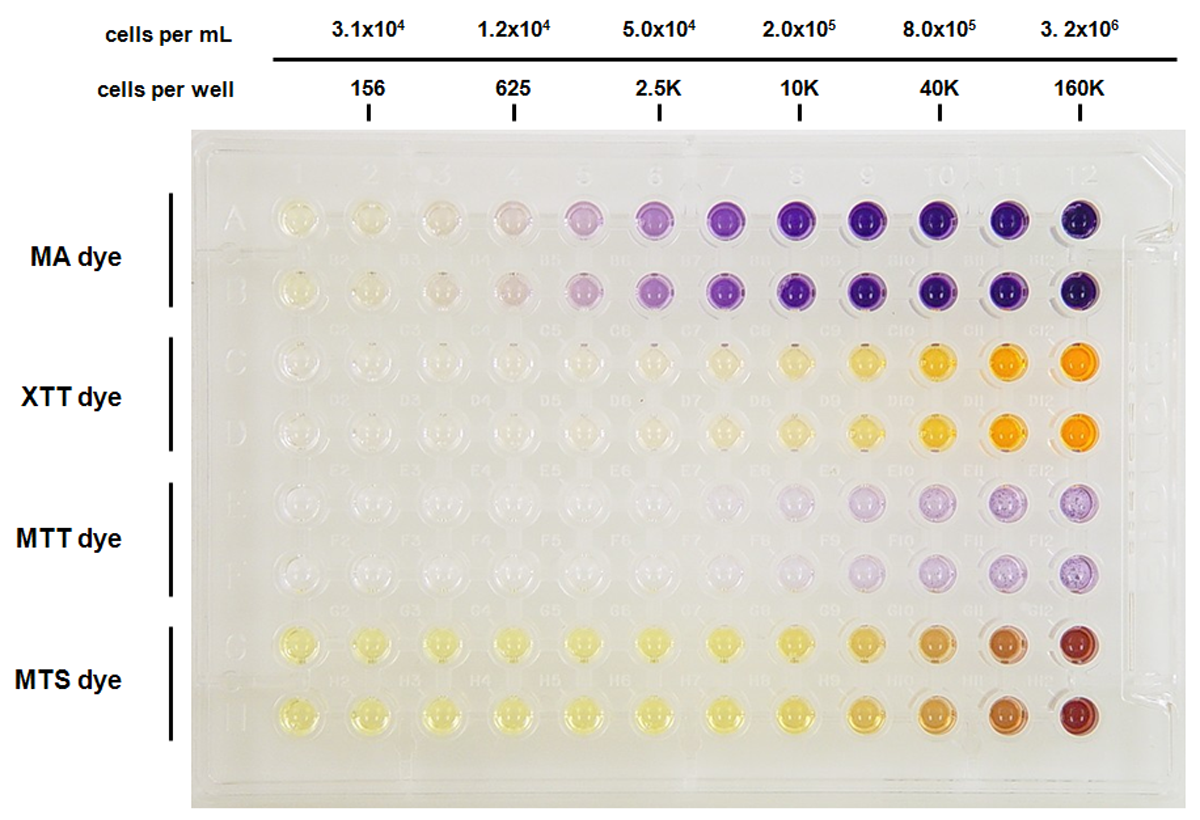

Supplement: Figure S1 — A549 lung cells were cultured as described in Materials and Methods , suspended in RPMI-1640 medium without phenol red, and 50 µL of cell suspension was added to each column of wells at 2-fold dilutions (right to left) in the same medium. After 4 hours, 10 µL of 4 different redox dye chemistries were added to a final tetrazolium concentration of 500 µM. (TIF) [file pone.0018147.s001.tif]

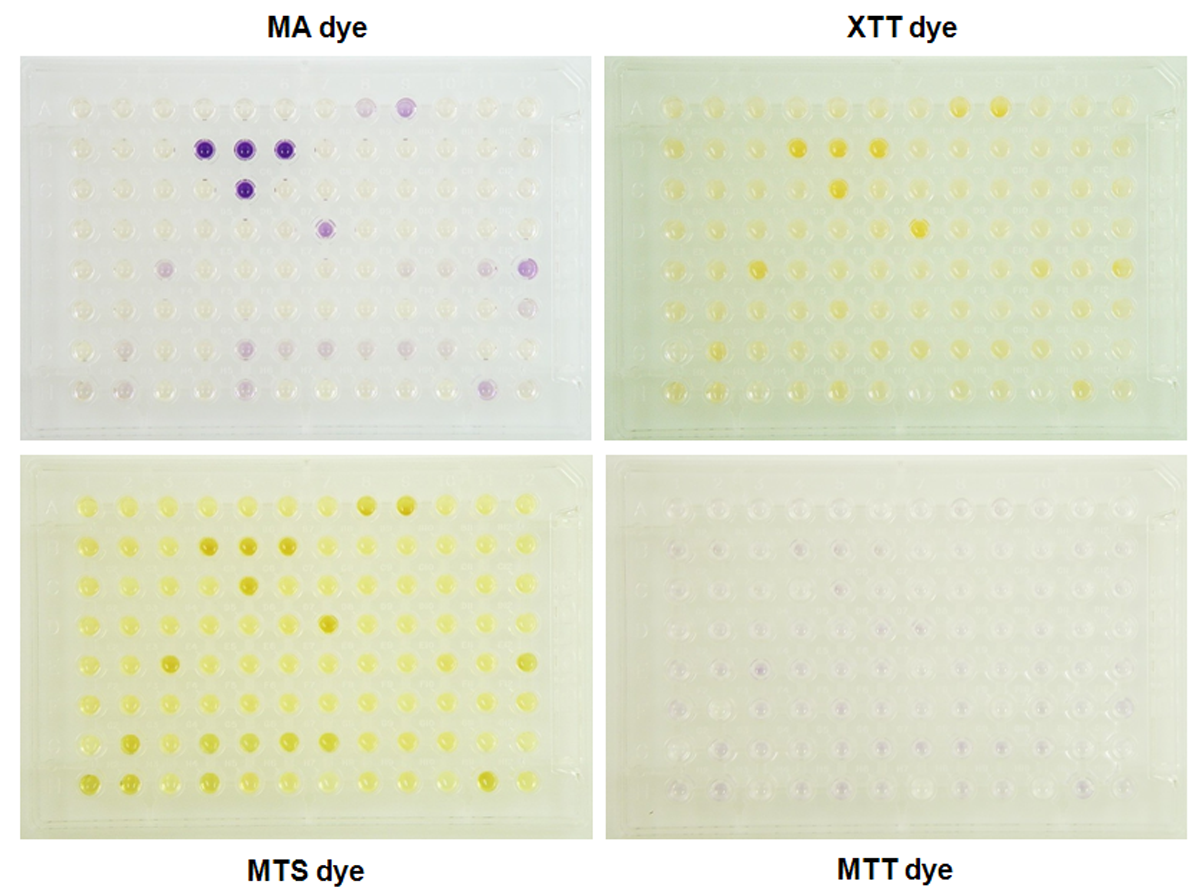

Supplement: Figure S2 — HepG2/C3A liver cells were cultured in Phenotype MicroArray PM-M1 for 40 hours and assayed for dye reduction, as described in Materials and Methods . Four different redox dye chemistries were used but in all cases, the concentration of the tetrazolium dye was 500 µM. (TIF) [file pone.0018147.s002.tif]

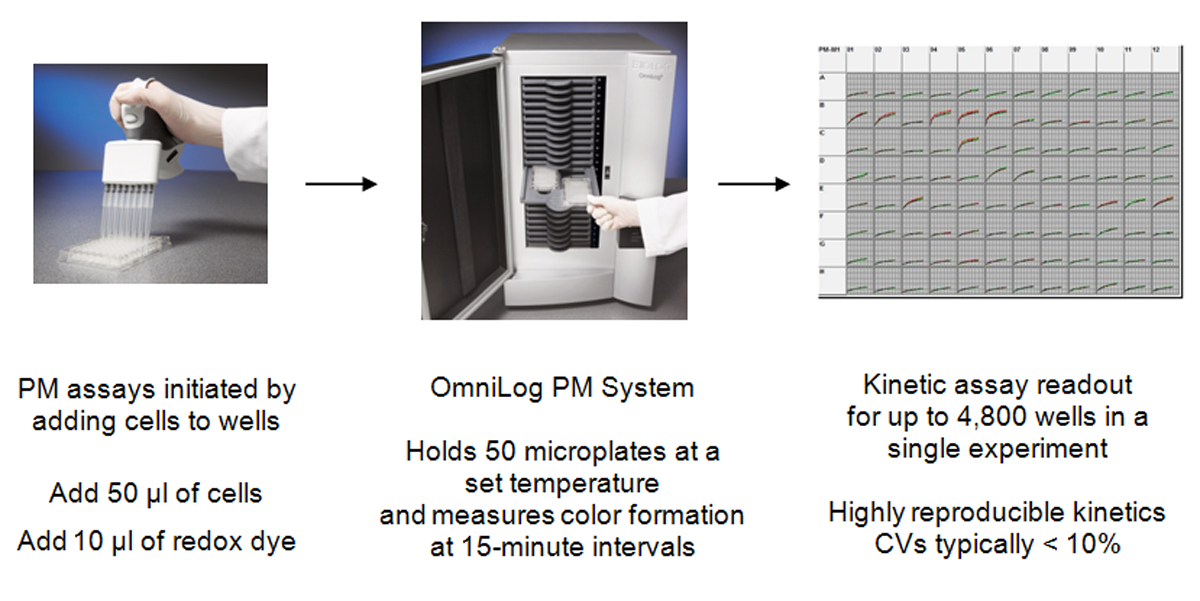

Supplement: Figure S3 — PM Assay with kinetics of dye reduction recorded by the OmniLog instrument. This figure shows the steps in running a PMM assay. Assays are initiated by adding a cell suspension to the wells, followed by addition of a redox dye. The PMM microplates are placed inside of the OmniLog instrument, which incubates the microplates and reads the color formation in wells with an internal color video camera every 15 minutes. The OmniLog software then generates kinetic graphs of color versus time for all wells. These assays have very high reproducibility. In the example shown, HME human breast cells (a generous gift of Dr. Chris Torrance, Horizon Discovery Ltd., Cambridge, UK) were tested using the standard protocol, but without serum and with the glutamine concentration increased to 2 mM. The graph shows a triplicate repeat of the assay with runs shown in green, red, and black. (TIF) [file pone.0018147.s003.tif]

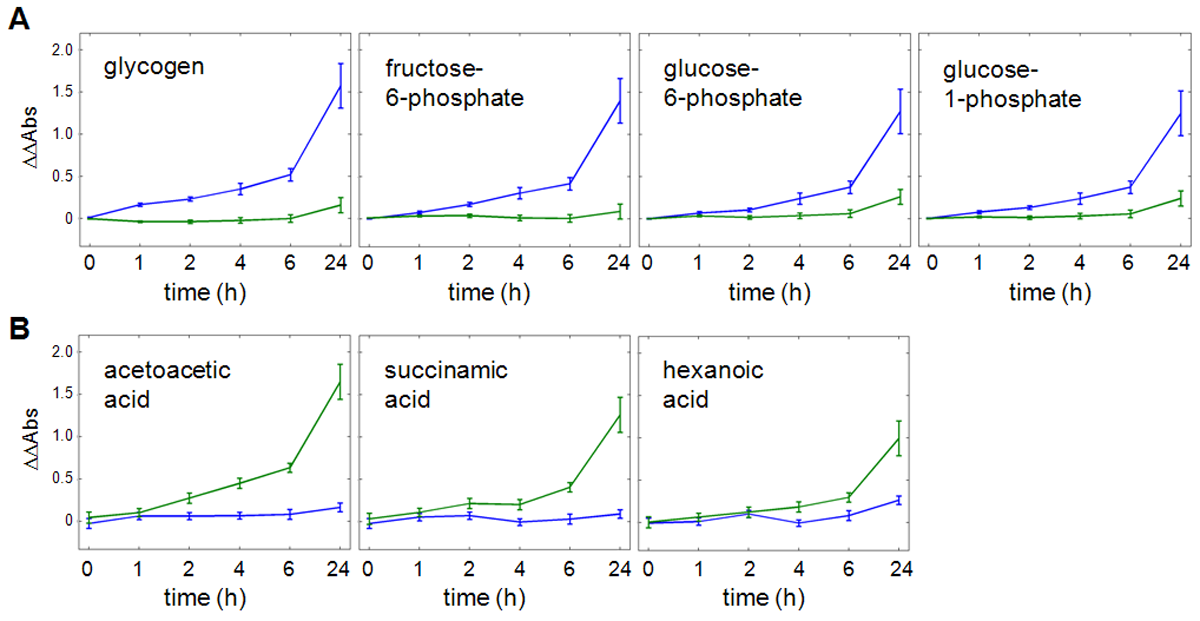

Supplement: Figure S4 — Comparison of substrate metabolism in brown and white preadipocytes and adipocytes. All adipoctye cell lines were assayed with Redox Dye Mix MB. (A) Substrates resulting in the greatest difference in comparison score (CS) at 24 hours between immortalized brown preadipocytes (blue lines) and 3T3-L1 white preadipocytes (green lines). To simplify the analysis, we only considered the CS at the 24-hour time point measurement, reasoning that since the accumulation of signal only increases over time, we would detect the most stable differences at this time, rather than only detecting kinetic changes in absorbance. (B) Substrates resulting in the greatest difference in CS at 24 hours between fully differentiated brown (blue lines) and 3T3-L1 white adipocytes (green lines). Graphs are depicted as the normalized change in absorbance (ΔΔAbs), using the zero-hour time point as baseline. (TIF) [file pone.0018147.s004.tif]

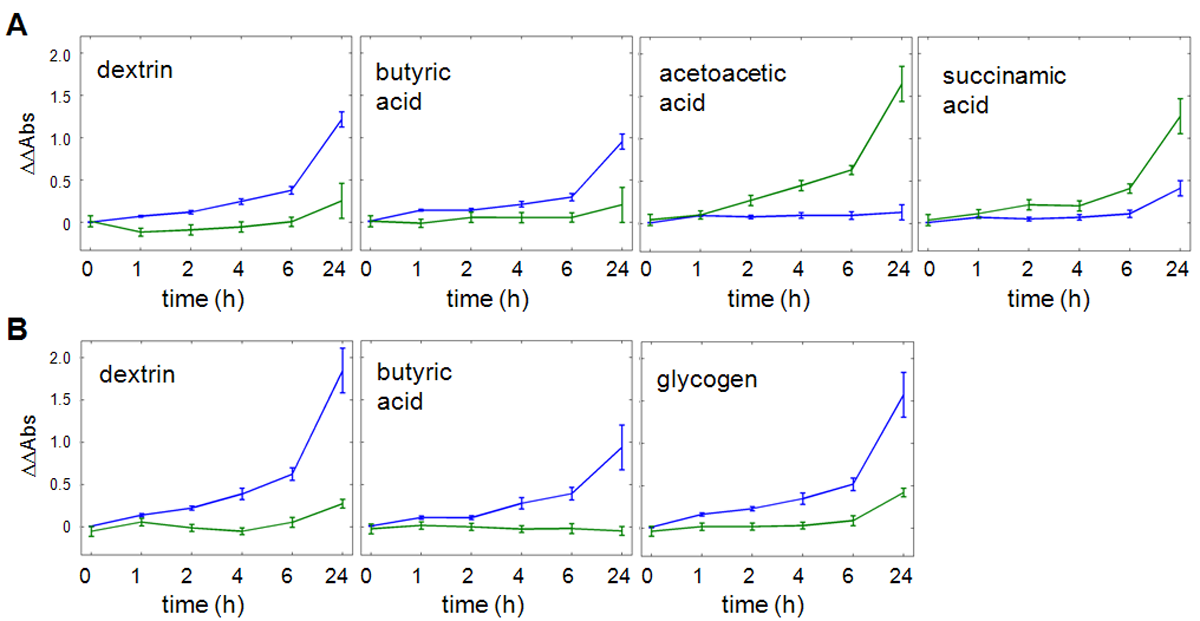

Supplement: Figure S5 — Analysis of effects of adipocyte differentiation on substrate metabolism. (A) Substrates resulting in the greatest difference in comparison score (CS) at 24 hours between undifferentiated 3T3-L1 preadipocytes (blue lines) and 3T3-L1 white adipocytes (green lines). (B) Substrates resulting in the greatest difference in CS at 24 hours between undifferentiated brown preadipocytes (blue lines) and brown adipocytes (green lines). Graphs are depicted as the normalized change in absorbance. (TIF) [file pone.0018147.s005.tif]
