# Supplementary material for: Assay of the Multiple Energy-Producing Pathways of Mammalian Cells
Source: PLoS One. 2011 Mar 24;6(3):e18147. doi: 10.1371/journal.pone.0018147 (PMC3063803; doi:10.1371/journal.pone.0018147)
Supplement: Table S1 — Plate maps of Phenotype MicroArray MicroPlates PM-M1 through -M4. (DOC) [file pone.0018147.s006.doc]

**Table S1.** Plate maps of Phenotype MicroArray MicroPlates PM-M1 through -M4

## PM-M1 MicroPlate™ - carbon-energy source assays

| **A1**  **Negative Control** | **A2**  **Negative Control** | **A3**  **Negative Control** | **A4**  **α-Cyclodextrin** | **A5**  **Dextrin** | **A6**  **Glycogen** | **A7**  **Maltitol** | **A8**  **Maltotriose** | **A9**  **D-Maltose** | **A10**  **D-Trehalose** | **A11**  **D-Cellobiose** | **A12**  **β-Gentiobiose** |
| --- | --- | --- | --- | --- | --- | --- | --- | --- | --- | --- | --- |
| **B1**  **D-Glucose-6-Phosphate** | **B2**  **α-D-Glucose-1-Phosphate** | **B3**  **L-Glucose** | **B4**  **α-D-Glucose** | **B5**  **α-D-Glucose** | **B6**  **α-D-Glucose** | **B7**  **3-O-Methyl-D-Glucose** | **B8**  **α-Methyl-D-Glucoside** | **B9**  **β-Methyl-D-Glucoside** | **B10**  **D-Salicin** | **B11**  **D-Sorbitol** | **B12**  **N-Acetyl-D-Glucosamine** |
| **C1**  **D-Glucosaminic Acid** | **C2**  **D-Glucuronic Acid** | **C3**  **Chondroitin-6-Sulfate** | **C4**  **Mannan** | **C5**  **D-Mannose** | **C6**  **α-Methyl-D-Mannoside** | **C7**  **D-Mannitol** | **C8**  **N-Acetyl-β-D-Mannosamine** | **C9**  **D-Melezitose** | **C10**  **Sucrose** | **C11**  **Palatinose** | **C12**  **D-Turanose** |
| **D1**  **D-Tagatose** | **D2**  **L-Sorbose** | **D3**  **L-Rhamnose** | **D4**  **L-Fucose** | **D5**  **D-Fucose** | **D6**  **D-Fructose-6-Phosphate** | **D7**  **D-Fructose** | **D8**  **Stachyose** | **D9**  **D-Raffinose** | **D10**  **D-Lactitol** | **D11**  **Lactulose** | **D12**  **α-D-Lactose** |
| **E1**  **Melibionic Acid** | **E2**  **D-Melibiose** | **E3**  **D-Galactose** | **E4**  **α-Methyl-D-Galactoside** | **E5**  **β-Methyl-D-Galactoside** | **E6**  **N-Acetyl-Neuraminic Acid** | **E7**  **Pectin** | **E8**  **Sedoheptulosan** | **E9**  **Thymidine** | **E10**  **Uridine** | **E11**  **Adenosine** | **E12**  **Inosine** |
| **F1**  **Adonitol** | **F2**  **L- Arabinose** | **F3**  **D-Arabinose** | **F4**  **β-Methyl-D-Xylopyranoside** | **F5**  **Xylitol** | **F6**  **Myo-Inositol** | **F7**  **Meso-Erythritol** | **F8**  **Propylene glycol** | **F9**  **Ethanolamine** | **F10**  **D,L- α-Glycerol-Phosphate** | **F11**  **Glycerol** | **F12**  **Citric Acid** |
| **G1**  **Tricarballylic Acid** | **G2**  **D,L-Lactic Acid** | **G3**  **Methyl D-lactate** | **G4**  **Methyl pyruvate** | **G5**  **Pyruvic Acid** | **G6**  **α-Keto-Glutaric Acid** | **G7**  **Succinamic Acid** | **G8**  **Succinic Acid** | **G9**  **Mono-Methyl Succinate** | **G10**  **L-Malic Acid** | **G11**  **D-Malic Acid** | **G12**  **Meso-Tartaric Acid** |
| **H1**  **Acetoacetic Acid (a)** | **H2**  **γ-Amino-N-Butyric Acid** | **H3**  **α-Keto-**  **Butyric Acid** | **H4**  **α-Hydroxy-Butyric Acid** | **H5**  **D,L-β-Hydroxy-Butyric Acid** | **H6**  **γ-Hydroxy-Butyric Acid** | **H7**  **Butyric Acid** | **H8**  **2,3-Butanediol** | **H9**  **3-Hydroxy-2-Butanone** | **H10**  **Propionic Acid** | **H11**  **Acetic Acid** | **H12**  **Hexanoic Acid** |

## PM-M2 MicroPlate™ - carbon-energy and nitrogen source assays

| **A1**  **Negative Control** | **A2**  **Negative Control** | **A3**  **Negative Control** | **A4**  **Tween 20** | **A5**  **Tween 40** | **A6**  **Tween 80** | **A7**  **Gelatin** | **A8**  **L-Alaninamide** | **A9**  **L-Alanine** | **A10**  **D-Alanine** | **A11**  **L-Arginine** | **A12**  **L-Asparagine** |
| --- | --- | --- | --- | --- | --- | --- | --- | --- | --- | --- | --- |
| **B1**  **L-Aspartic Acid** | **B2**  **D-Aspartic Acid** | **B3**  **L-Glutamic Acid** | **B4**  **D-Glutamic Acid** | **B5**  **L-Glutamine** | **B6**  **Glycine** | **B7**  **L-Histidine** | **B8**  **L-Homoserine** | **B9**  **Hydroxy-L-Proline** | **B10**  **L-Isoleucine** | **B11**  **L-Leucine** | **B12**  **L-Lysine** |
| **C1**  **L-Methionine** | **C2**  **L-Ornithine** | **C3**  **L-Phenylalanine** | **C4**  **L-Proline** | **C5**  **L-Serine** | **C6**  **D-Serine** | **C7**  **L-Threonine** | **C8**  **D-Threonine** | **C9**  **L-Tryptophan** | **C10**  **L-Tyrosine** | **C11**  **L-Valine** | **C12**  **Ala-Ala** |
| **D1**  **Ala-Arg** | **D2**  **Ala-Asn** | **D3**  **Ala-Asp** | **D4**  **Ala-Glu** | **D5**  **Ala-Gln** | **D6**  **Ala-Gly** | **D7**  **Ala-His** | **D8**  **Ala-Ile** | **D9**  **Ala-Leu** | **D10**  **Ala-Lys** | **D11**  **Ala-Met** | **D12**  **Ala-Phe** |
| **E1**  **Ala-Pro** | **E2**  **Ala-Ser** | **E3**  **Ala-Thr** | **E4**  **Ala-Trp** | **E5**  **Ala-Tyr** | **E6**  **Ala-Val** | **E7**  **Arg-Ala**  **(b)** | **E8**  **Arg-Arg**  **(b)** | **E9**  **Arg-Asp** | **E10**  **Arg-Gln** | **E11**  **Arg-Glu** | **E12**  **Arg-Ile**  **(b)** |
| **F1**  **Arg-Leu**  **(b)** | **F2**  **Arg-Lys**  **(b)** | **F3**  **Arg-Met**  **(b)** | **F4**  **Arg-Phe**  **(b)** | **F5**  **Arg-Ser**  **(b)** | **F6**  **Arg-Trp** | **F7**  **Arg-Tyr**  **(b)** | **F8**  **Arg-Val**  **(b)** | **F9**  **Asn-Glu** | **F10**  **Asn-Val** | **F11**  **Asp-Ala** | **F12**  **Asp-Asp** |
| **G1**  **Asp-Glu** | **G2**  **Asp-Gln** | **G3**  **Asp-Gly** | **G4**  **Asp-Leu** | **G5**  **Asp-Lys** | **G6**  **Asp-Phe** | **G7**  **Asp-Trp** | **G8**  **Asp-Val** | **G9**  **Glu-Ala** | **G10**  **Glu-Asp** | **G11**  **Glu-Glu** | **G12**  **Glu-Gly** |
| **H1**  **Glu-Ser** | **H2**  **Glu-Trp** | **H3**  **Glu-Tyr** | **H4**  **Glu-Val** | **H5**  **Gln-Glu** | **H6**  **Gln-Gln** | **H7**  **Gln-Gly** | **H8**  **Gly-Ala** | **H9**  **Gly-Arg** | **H10**  **Gly-Asn** | **H11**  **Gly-Asp** | **H12**  **α-D-Glucose** |

## PM-M3 MicroPlate™ - carbon-energy and nitrogen source assays

| **A1**  **Negative Control** | **A2**  **Negative Control** | **A3**  **Negative Control** | **A4**  **Gly-Gly** | **A5**  **Gly-His** | **A6**  **Gly-Ile** | **A7**  **Gly-Leu** | **A8**  **Gly-Lys** | **A9**  **Gly-Met** | **A10**  **Gly-Phe** | **A11**  **Gly-Pro** | **A12**  **Gly-Ser** |
| --- | --- | --- | --- | --- | --- | --- | --- | --- | --- | --- | --- |
| **B1**  **Gly-Thr** | **B2**  **Gly-Trp** | **B3**  **Gly-Tyr** | **B4**  **Gly-Val** | **B5**  **His-Ala** | **B6**  **His-Asp** | **B7**  **His-Glu** | **B8**  **His-Gly** | **B9**  **His-His**  **(c)** | **B10**  **His-Leu** | **B11**  **His-Lys**  **(d)** | **B12**  **His-Met** |
| **C1**  **His-Pro** | **C2**  **His-Ser** | **C3**  **His-Trp** | **C4**  **His-Tyr** | **C5**  **His-Val** | **C6**  **Ile-Ala** | **C7**  **Ile-Arg**  **(b)** | **C8**  **Ile-Asn** | **C9**  **Ile-Gln** | **C10**  **Ile-Gly** | **C11**  **Ile-His** | **C12**  **Ile-Ile** |
| **D1**  **Ile-Leu** | **D2**  **Ile-Met** | **D3**  **Ile-Phe** | **D4**  **Ile-Pro** | **D5**  **Ile-Ser** | **D6**  **Ile-Trp** | **D7**  **Ile-Tyr** | **D8**  **Ile-Val** | **D9**  **Leu-Ala** | **D10**  **Leu-Arg**  **(b)** | **D11**  **Leu-Asn** | **D12**  **Leu-Asp** |
| **E1**  **Leu-Glu** | **E2**  **Leu-Gly** | **E3**  **Leu-His** | **E4**  **Leu-Ile** | **E5**  **Leu-Leu** | **E6**  **Leu-Met** | **E7**  **Leu-Phe** | **E8**  **Leu-Pro** | **E9**  **Leu-Ser** | **E10**  **Leu-Trp** | **E11**  **Leu-Tyr** | **E12**  **Leu-Val** |
| **F1**  **Lys-Ala**  **(d)** | **F2**  **Lys-Arg**  **(b)** | **F3**  **Lys-Asp** | **F4**  **Lys-Glu** | **F5**  **Lys-Gly** | **F6**  **Lys-Ile**  **(b)** | **F7**  **Lys-Leu**  **(b)** | **F8**  **Lys-Lys** | **F9**  **Lys-Met**  **(e)** | **F10**  **Lys-Phe** | **F11**  **Lys-Pro** | **F12**  **Lys-Ser** |
| **G1**  **Lys-Thr** | **G2**  **Lys-Trp**  **(b)** | **G3**  **Lys-Tyr**  **(b)** | **G4**  **Lys-Val**  **(d)** | **G5**  **Met-Arg**  **(b)** | **G6**  **Met-Asp** | **G7**  **Met-Gln** | **G8**  **Met-Glu** | **G9**  **Met-Gly** | **G10**  **Met-His** | **G11**  **Met-Ile** | **G12**  **Met-Leu** |
| **H1**  **Met-Lys**  **(e)** | **H2**  **Met-Met** | **H3**  **Met-Phe** | **H4**  **Met-Pro** | **H5**  **Met-Thr** | **H6**  **Met-Trp** | **H7**  **Met-Tyr** | **H8**  **Met-Val** | **H9**  **Phe-Ala** | **H10**  **Phe-Asp** | **H11**  **Phe-Glu** | **H12**  **α-D-Glucose** |

## PM-M4 MicroPlate™ - carbon-energy and nitrogen source assays

| **A1**  **Negative Control** | **A2**  **Negative Control** | **A3**  **Negative Control** | **A4**  **Phe-Gly** | **A5**  **Phe-Ile** | **A6**  **Phe-Met** | **A7**  **Phe-Phe** | **A8**  **Phe-Pro** | **A9**  **Phe-Ser** | **A10**  **Phe-Trp** | **A11**  **Phe-Tyr** | **A12**  **Phe-Val** |
| --- | --- | --- | --- | --- | --- | --- | --- | --- | --- | --- | --- |
| **B1**  **Pro-Ala** | **B2**  **Pro-Arg**  **(b)** | **B3**  **Pro-Asn** | **B4**  **Pro-Asp** | **B5**  **Pro-Glu** | **B6**  **Pro-Gln** | **B7**  **Pro-Gly** | **B8**  **Pro-Hyp** | **B9**  **Pro-Ile** | **B10**  **Pro-Leu** | **B11**  **Pro-Lys**  **(b)** | **B12**  **Pro-Phe** |
| **C1**  **Pro-Pro** | **C2**  **Pro-Ser** | **C3**  **Pro-Trp** | **C4**  **Pro-Tyr** | **C5**  **Pro-Val** | **C6**  **Ser-Ala** | **C7**  **Ser-Asn** | **C8**  **Ser-Asp** | **C9**  **Ser-Glu** | **C10**  **Ser-Gln** | **C11**  **Ser-Gly** | **C12**  **Ser-His**  **(b)** |
| **D1**  **Ser-Leu** | **D2**  **Ser-Met** | **D3**  **Ser-Phe** | **D4**  **Ser-Pro** | **D5**  **Ser-Ser** | **D6**  **Ser-Tyr** | **D7**  **Ser-Val** | **D8**  **Thr-Ala** | **D9**  **Thr-Arg**  **(f)** | **D10**  **Thr-Asp** | **D11**  **Thr-Glu** | **D12**  **Thr-Gln** |
| **E1**  **Thr-Gly** | **E2**  **Thr-Leu** | **E3**  **Thr-Met** | **E4**  **Thr-Phe** | **E5**  **Thr-Pro** | **E6**  **Thr-Ser** | **E7**  **Trp-Ala** | **E8**  **Trp-Arg** | **E9**  **Trp-Asp** | **E10**  **Trp-Glu** | **E11**  **Trp-Gly** | **E12**  **Trp-Leu** |
| **F1**  **Trp-Lys**  **(e)** | **F2**  **Trp-Phe** | **F3**  **Trp-Ser** | **F4**  **Trp-Trp** | **F5**  **Trp-Tyr** | **F6**  **Trp-Val** | **F7**  **Tyr-Ala** | **F8**  **Tyr-Gln** | **F9**  **Tyr-Glu** | **F10**  **Tyr-Gly** | **F11**  **Tyr-His** | **F12**  **Tyr-Ile** |
| **G1**  **Tyr-Leu** | **G2**  **Tyr-Lys** | **G3**  **Tyr-Phe** | **G4**  **Tyr-Trp** | **G5**  **Tyr-Tyr** | **G6**  **Tyr-Val** | **G7**  **Val-Ala** | **G8**  **Val-Arg** | **G9**  **Val-Asn** | **G10**  **Val-Asp** | **G11**  **Val-Glu** | **G12**  **Val-Gln** |
| **H1**  **Val-Gly** | **H2**  **Val-His** | **H3**  **Val-Ile** | **H4**  **Val-Leu** | **H5**  **Val-Lys** | **H6**  **Val-Met** | **H7**  **Val-Phe** | **H8**  **Val-Pro** | **H9**  **Val-Ser** | **H10**  **Val-Tyr** | **H11**  **Val-Val** | **H12**  **α-D-Glucose** |
